# European and American Strains of Porcine Parainfluenza Virus 1 (PPIV-1) Belong to Two Distinct Genetic Lineages

**DOI:** 10.3390/pathogens11030375

**Published:** 2022-03-20

**Authors:** Tomasz Stadejek, Piotr Cybulski, Phillip C. Gauger, Aleksandra Woźniak

**Affiliations:** 1 Department of Pathology and Veterinary Diagnostics, Institute of Veterinary Medicine, Warsaw University of Life Sciences - SGGW, Nowoursynowska 159C, 02-776 Warsaw, Poland; 2 Goodvalley Agro S.A., Dworcowa 25, 77-320 Przechlewo, Poland; piotr.cybulski.dvm@gmail.com; 3 Veterinary Diagnostic and Production Animal Medicine, Iowa State University College of Veterinary Medicine, 1800 Christensen Drive Ames, IA 50011-1134, USA; pcgauger@iastate.edu

**Keywords:** PPIV-1, PRV-1, parainfluenza, sequencing, epidemiology

## Abstract

Porcine parainfluenza virus 1 (PPIV-1) is a recently emerged respirovirus closely related to human parainfluenza virus 1 (HPIV-1) and Sendai virus (SenV). PPIV-1 has been detected in Asia, the Americas and Europe, but knowledge on its epidemiology and genetic diversity is very limited. In the present study, the complete nucleotide sequences of the fusion (F)-protein gene obtained from samples from 12 Polish and 11 US herds were analysed and compared to previously available genetic data from the Americas, Asia and Europe. The existence of two distinct clades was observed, grouping European sequences and one Hong Kong sequence (clade 1), or one American sequence and three Asian sequences (clade 2). The mean genetic distances measured with the p-distance were 0.04 (S.E., 0.000) within both clades, and 0.095 (S.E., 0.006) between the clades. Moreover, two distinct clusters of highly similar sequences were identified, which corresponded to the geographically distant nurseries and finishing units, from three pig flows within one Polish pig-production company. The obtained data indicate that the two PPIV-1 lineages may have evolved independently in Europe and America. More studies, particularly involving Asian viruses, are necessary to understand the virus’ emergence and epidemiology and the role of carriers in the spread of PPIV-1.

## 1. Introduction

The genus respirovirus belongs to the family *Paramyxoviridae*, order Mononegavirales, and includes six species causing respiratory tract infections of humans and animals: *bovine respirovirus 3* (virus name: bovine parainfluenza virus 3—BPIV-3), *caprine respirovirus 3* (virus name: caprine parainfluenza virus 3—CPIV-3), *human respirovirus 1* (virus name: human parainfluenza virus 1—HPIV-1), *human respirovirus 3* (virus name: human parainfluenza virus 3—HPIV-3), *murine respirovirus 1* (virus name: Sendai virus—SenV) and the most recently discovered *porcine respirovirus 1* (virus name: porcine parainfluenza virus 1—PPIV-1) [1]. 

Porcine parainfluenza virus 1 was first detected in samples from 2009 in Hong Kong, and later in samples from 2015 to 2020 in the USA, Hungary, Chile, the Netherlands, Germany and Poland [2,3,4,5,6,7,8]. Therefore, it can safely be assumed that the virus is widely distributed globally. It has been proposed to contribute to multifactorial respiratory disease, and can cause coughing, sneezing and nasal discharge [3]. 

The analysis of PPIV-1 nucleotide sequences indicated a certain level of genetic diversity of the virus [3,4,5,6,7]. However, very few sequences are publicly accessible, and no genetic data from most pig-producing countries is available. Therefore, the evolution of the pathogen and its molecular epidemiology are unknown. Previous studies concluded that gene analyses of the fusion (F) protein, RdRp polymerase (L) or hemagglutinin-neuraminidase (H-N) provided phylogenetic clustering similar to the one observed following complete or nearly complete genome analysis [7]. Thus, individual gene analysis seems to be sufficient and cost effective for PPIV-1 genotyping. The most recent study describing the complete sequence of a German PPIV-1 strain showed that the L- and F-protein gene sequences of Hungarian, German and Hong Kong viruses were clustered separately from North American, Chilean and Chinese viruses and others from Hong Kong [7].

The F protein is a type I glycoprotein, with a cleaved N-terminal hydrophobic signal peptide and a C-proximal membrane anchor. It mediates the penetration of the host cell by the fusion of the viral envelope to the plasma membrane. The F protein is synthesised as an inactive precursor, F_0_, which is cleaved to the two subunits: the smaller F_2_ and larger F_1_, which contains a membrane anchor. The cleavage of F_0_ is a prerequisite for paramyxovirus infectivity [9]. 

The aim of the study was to analyse and compare the genetic diversity of F-gene sequences from Poland and the USA and to provide better insights into the global PPIV-1 epidemiology. 

## 2. Results

**Figure 1:**
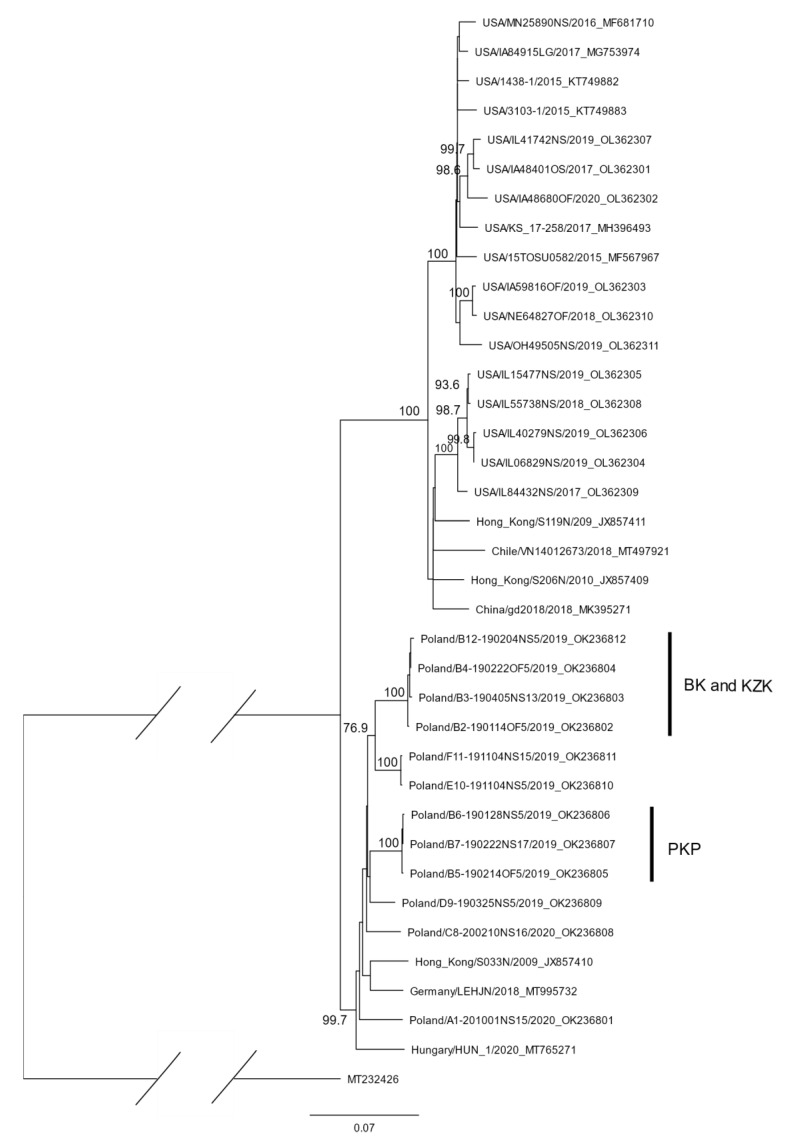
Maximum-likelihood phylogenetic tree constructed from 36 nucleotide sequences of F gene of PPIV-1. The analysis was performed with the Geneious 10.2.6 computer program, using MUSCLE alignment and PhyML with Jukes–Cantor substitution model, and 1000 replicates. Only bootstrap values ≥75 are shown. HPIV-1 (GenBank acc.: MT232426) F-gene sequence was used as an outgroup. Vertical bars correspond to clusters of sequences obtained from herds belonging to three different pig flows (BK, KZK and PKP) within production company B.

In this study, 23 complete F-gene sequences of PPIV-1 strains from 12 Polish and 11 US swine herds were obtained (Table 1). All the sequences were 1674 nucleotides long with no indels. Their analysis, together with the sequences previously available in GenBank, showed their grouping in two distinct clades with high statistical support (Figure 1). One clade contained 12 sequences from Poland, one from Hungary, one from Germany and one from Hong Kong (clade 1), and the other one, 17 American, one Chilean, one Chinese and two Hong Kong sequences (clade 2). The identities of the nucleotide sequences within the two clades ranged from 93.2% to 99.9% for clade 2, and from 94.6% to 99.9% for clade 1. The identity of the sequences between the clades ranged from 88.7% to 91.9%. The mean genetic distances measured with *p*-distance were 0.04 (S.E., 0.000) within both clades, and 0.095 (S.E., 0.006) between the clades. The identity of the amino acid sequences within the clades was similar to that of the nucleotide sequences, and the high statistical support for the two clades was maintained (data not shown). Amino acid substitutions were scattered along the sequences, being slightly more frequent in the amino- and carboxy-termini. These fragments in other paramyxoviruses contain cleaved amino-terminal signal peptides and carboxy-terminal transmembrane domains and cytoplasmic tails [9]. Similarly, to the closely related HPIV-1 and SeV, all the analysed sequences of PPIV-1 lacked a furine F_0_ cleavage motif and the single-basic cleavage site P-Q-Y-R↓F between them [2].

## 3. Discussion

The data presented in the current study provide the first solid evidence of the existence of the divergent evolution of at least two genetic lineages of PPIV-1, mainly European lineage 1 and mainly American lineage 2. The current results are in line with the previous observations of a separate grouping of the sequences from Hungary and Germany, away from American sequences [4,5,6,7]. It should be emphasised that the oldest PPIV-1 sequences known to date are from Hong Kong from 2009 and 2010, and belonged to both lineages [2]. It is intriguing that, in a relatively small pig-production area of Hong Kong, PPIV-1 strains of both lineages already co-existed in 2009–2010, several years before the discovery of the virus in the USA and Europe. It remains to be verified whether the genetic diversity of the PPIV-1 sequences in Hong Kong reflects the epidemiological situation in mainland China and the rest of eastern Asia. Importantly, the F_0_ cleavage site, which, in some paramyxoviruses, is related to the level of virulence, was conserved between all the PPIV-1 sequences analysed.

Currently, paramyxovirus species demarcation is based on the distance in the phylogenetic tree of complete L-protein amino acid sequences. The classification into genotypes and clades for epidemiological purposes differs between the species. The three examples of respiroviruses with well-studied within-species genetic diversity are HPIV-1, HPIV-3 and BPIV-3. In HPIV-1, three clades (1, 2 and 3) were proposed based on the HN gene [10]. The mean within-clade *p*-distance was determined to be 0.0022 to 0.0109, and the between-clade *p*-distance was between 0.0192 and 0.0368 [10]. In HPIV-3, six clusters (A, B, C1, C2, C3 and C5) were defined, with the mean within-cluster *p*-distance ranging from 0.0066 to 0.0325, and a between-cluster *p*-distance between 0.0262 and 0.0665 [10]. BPIV-3, apparently being more genetically diverse than the two aforementioned species, is classified in three genotypes—A, B and C—with about 81–82% identity for the F-gene sequences between them [11]. In comparison, the genetic distance between the two known lineages of PPIV-1 seems to be higher than that of HPIV-1 clades and HPIV-3 clusters, and lower than that of BPIV-3 genotypes. A more precise definition of PPIV-1 clades or genotypes will require the sequencing of different genomic regions of more viruses from more countries and geographic regions.

As the majority of the available PPIV-1 sequences originate from Europe and the USA, it is difficult to conclude when the two virus lineages emerged and their global prevalence. In the case of BPIV-3, it is known that genotype A is prevalent worldwide; B, in Australia, Argentina and the USA; and C, in Asia and the USA [12]. However, Kumagai et al. (2020) reported that the genetic diversity of BPIV-3 in Japan does not reflect the international cattle trade patterns of the country [12]. Surprisingly, it was found to be different from that of the USA and Australia, from where cattle was imported to Japan, but similar to that of China and Korea, with which no live animal trade exists. 

A closer analysis of the PPIV-1 clades consisting of the lineage 1 and lineage 2 sequences provided some hints of the role of live pig movements in the virus’ spread. For example, three and four sequences from seven geographically distant locations in Poland were positioned in two clusters of highly identical sequences (Figure 1). Interestingly, all of them belonged to the same production company (all farms B; see Table 1). Further analysis of the composition of the two sequence clusters from farms B, and the links between them in the company pig flows, showed highly identical sequences in piglets from the gilt-producing herd B6, piglets from the nursery herd B5, and fatteners from the finishing herd B7 (flow PKP). Two other flows consisting of the gilt-producing herd B4, and nursery herd B12 (flow KZK), as well as nursery B2 and finishing herd B3 (flow BK), also shared highly identical viruses (Figure 1). The sequences from the two independent family farms E10 and F11 were nearly identical, too, and in this case, it might be explained by the fact that they neighbour each other. However, no information about direct links, such as pig or personnel movements between them, was available.

In lineage 2, the viruses from the USA belonged to two well-supported clusters consisting of 12 sequences from several Midwestern states and five sequences from Illinois, respectively (Figure 1). Only in the case of two farms (represented by the sequences USA/IL06829NS/2019 and USA/IL40279NS/2019) from the latter cluster could the high identity of the F gene be explained by the same ownership and, probably, the transportation of pigs between these farms or from a common source. More detailed analysis of the pig-trade patterns in the Midwest might explain the observed PPIV-1-diversity picture in the USA.

The finding of highly identical sequences specific for a given pig flow in company B may indicate that either the PPIV-1 infection can be long lasting on a population level, and pigs infected at an early age (e.g., having a low level of maternal immunity against PPIV-1) may shed the virus for several weeks and eventually infect penmates that avoided the infection at a very early age (e.g., due to a high level of maternal immunity), or that the PPIV-1 infection may be chronic. 

The role of maternal immunity in the protection against PPIV-1 infection is unknown. In our recent study, the virus was detected in nasal swabs of 5-week-old pigs from 15 out of 18 farms, which indicates that, in some herds, it plays a marginal role, while in others, it may provide solid protection against the infection [8]. Welch et al. (2021) showed that low levels of maternal antibodies in piglets challenged with PPIV-1 were not protective and the animals shed the virus with nasal excretion at least until 21 days post-inoculation, until the end of the experiment [13]. In theory, the length of shedding might be even longer, which may also help to sustain the infection in a population with diverse levels of immunity against the virus. Indeed, Woźniak et al. (2022) showed that, in some Polish farrow-to-finish pig farms, the virus was detected in most of the age groups tested, from 5 to more than 17 weeks old [8].

In summary, the current data, despite mostly being available from the USA and Poland, indicate that the two PPIV-1 lineages may have evolved independently in Europe and North America, and they might have spread to Asia, probably with infected animals. However, the experiences from the studies on the epidemiology of the related respiroviruses, such as HPIV-1, HPIV-3 and BPIV-3, suggest that the genetic diversity of PPIV-1 might be even higher. More studies, particularly involving Asian viruses, are necessary to understand the virus’ emergence and epidemiology and the role of carriers in the spread of PPIV-1.

## 4. Materials and Methods

### 4.1. Samples

The samples from Poland were nasal swabs and oral fluids from farms reporting mild-to-moderate influenza-like clinical signs, and were collected in 2019–2020 as part of an influenza A virus (IAV) surveillance study. The samples originated from 12 farms, 7 of which belonged to a single company (Table 1) [8]. The samples that tested positive in real-time RT-PCR with Ct < 30 [14] were used in RT-PCR in order to generate amplicons encompassing the whole F gene of PPIV-1, which were then sequenced with the Sanger method (Supplementary Table S1). The obtained DNA sequences were assembled with Geneious 10.2.6 (Biomatters Ltd., Auckland, New Zealand). 

The samples from the USA were collected for different diagnostic purposes, and involved nasal swabs, oral fluids, and a single oropharyngeal swab, from nursery pigs and one neonatal piglet. The samples originated from 11 production sites. With the exception of two farms, they belonged to different owners (Table 1). The samples were sequenced with the Sanger method using amplification and sequencing primers as previously described [4]. The F-gene nucleotide sequences were assembled with Geneious 11.1.5 (Biomatters Ltd., Auckland, New Zealand). The sequences obtained in this study are available in GenBank under accession numbers OK236801-OK236812 and OL362301-362311 (Table 1).

### 4.2. Phylogenetic Analyses

For sequence analysis, 17 complete or nearly complete F-gene nucleotide sequences from the USA, Hong Kong, China, Hungary, Chile and Germany previously published and available in GenBank on 15 January 2022 were downloaded. As the three sequences from a single Hungarian farm were nearly identical, only one representative sequence was kept for the analysis [5].

The sequence analysis was performed with Geneious 10.2.6. The sequences were aligned using MUSCLE [15], and a maximum-likelihood phylogenetic tree was reconstructed with PhyML using the Jukes–Cantor substitution model [16]. The reliability of the branching order was estimated by performing 1000 replicates, with values ≥ 75 defined as well supported [17]. The HPIV-1 (GenBank acc.: MT232426) F-gene sequence was used as an outgroup. 

The mean genetic distances within and between major clades of PPIV-1 were calculated with MEGA11 using the *p*-distance [18]. The distances are described in terms of the mean and standard error as determined using the bootstrap method with 1000 replicates.

Additionally, the nucleotide sequences were translated and aligned using MUSCLE, and a maximum-likelihood phylogenetic tree was reconstructed with PhyML using the Blosum62 substitution model, with 1000 replicates [16].

## Figures and Tables

**Table 1:** The details of sequences obtained in this study along with the complete description of the geographical origin, the age of pigs, their clinical signs and the sample type.

Farm ID	Virus Name	Province (PL)/ State (USA)	Age (Weeks)	Clinical Signs	Sample Type	GenBank No.
PL-A1	PL/A1-201001NS15/2020	Lubusz	15	Respiratory	Nasal swab	OK236801
PL-B2	PL/B2-190114OF5/2019	West Pomerania	5	Respiratory	Oral fluid	OK236802
PL-B3	PL/B3-190405NS13/2019	Greater Poland	13	None	Nasal swab	OK236803
PL-B4	PL/B4-190222OF5/2019	Pomerania	5	Respiratory	Oral fluid	OK236804
PL-B5	PL/B5-190214OF5/2019	Greater Poland	5	Respiratory	Oral fluid	OK236805
PL-B6	PL/B6-190128NS5/2019	Pomerania	5	Respiratory	Nasal swab	OK236806
PL-B7	PL/B7-190222NS17/2019	Pomerania	17	None	Nasal swab	OK236807
PL-B12	PL/B12-190204NS5/2019	Pomerania	5	Respiratory	Nasal swab	OK236812
PL-C8	PL/C8-200210NS16/2020	Kuyavia-Pomerania	16	Respiratory	Nasal swab	OK236808
PL-D9	PL/D9-190325NS5/2019	Silesia	5	Respiratory	Nasal swab	OK236809
PL-E10	PL/E10-191104NS5/2019	Lodz	5	Respiratory	Nasal swab	OK236810
PL-F11	PL/F11-191104NS15/2019	Lodz	15	Respiratory	Nasal swab	OK236811
US-G1	USA/IA48401OS/2017	Iowa	3	Unknown	Oropharyngeal swab	OL362301
US-E2	USA/IL84432NS/2017	Illinois	Unknown	Unknown	Nasal swab	OL362309
US-H3	USA/IL55738NS/2018	Illinois	3 days	Unknown	Nasal swab	OL362308
US-I4	USA/NE64827OF/2018	Nebraska	Unknown	Unknown	Oral fluid	OL362310
US-J5	USA/IL06829NS/2019	Illinois	3	Unknown	Nasal swab	OL362304
US-K6	USA/IL15477NS/2019	Illinois	3	Unknown	Nasal swab	OL362305
US-J7	USA/IL40279NS/2019	Illinois	3	Unknown	Nasal swab	OL362306
US-L8	USA/IL41742NS/2019	Illinois	3	Respiratory	Nasal swab	OL362307
US-M9	USA/OH49505NS/2019	Ohio	3	Unknown	Nasal swab	OL362311
US-N10	USA/IA59816OF/2019	Iowa	5	Unknown	Oral fluid	OL362303
US-O11	USA/IA48680OF/2020	Iowa	4	Unknown	Oral fluid	OL362302

## Data Availability

The data presented in this study are available on request. The sequences obtained in this study are available in GenBank under accession numbers OK236801-OK236812 and OL362301-362311.

## Supplementary Materials

The following supporting information can be downloaded at: https://www.mdpi.com/article/10.3390/pathogens11030375/s1. Supplementary Table S1: Sequencing primers used for sequencing of F gene of Polish PPIV-1 viruses.
