# Supplementary material for: European and American Strains of Porcine Parainfluenza Virus 1 (PPIV-1) Belong to Two Distinct Genetic Lineages
Source: Pathogens. 2022 Mar 20;11(3):375. doi: 10.3390/pathogens11030375 (PMC8948755; doi:10.3390/pathogens11030375)
Supplement: Supplementary file 1 [file pathogens-11-00375-s001.zip › pathogens-1622828-supplementary.pdf]

Supplementary table S1. Sequencing primers used for sequencing of F gene of Polish PPIV-1 viruses.

Primer	Nucleotide position in PPIV-1 genome (MT995732)	Sequence	Reference
F-For1	4827-4852	ACTTAGGGTACAAGTTATCCAAAAAA	Park et al. 2019 [4]
F-For2	5537-5561	GAGAGAAGAGCTTAACATTACAGGC	Park et al. 2019 [4]
F-For3	5338-5361	TAAAAGAAGCAATGGAGAAGACCC	This work
F-For4	5376-5398	GATACTGGCTCTCAAAATGCTCC	This work
F-Rev1	5611-5636	TCATAAATATCTGTYTTCCCGAGATT	Park et al. 2019 [4]
F-Rev2	6581-6600	TCGTGCACCCTAAGTTTTCTTTA	Park et al. 2019 [4]
